# Identification of Novel Characteristics in TP53-Mutant Hepatocellular Carcinoma Using Bioinformatics

**DOI:** 10.3389/fgene.2022.874805

**Published:** 2022-05-16

**Authors:** Yang Yang, Yajuan Qu, Zhaopeng Li, Zhiyong Tan, Youming Lei, Song Bai

**Affiliations:** ^1^ The First Affiliated Hospital of Kunming Medical University, Kunming, China; ^2^ Department of Rehabilitation Medicine, Qujing Second People's Hospital, Qujing, China; ^3^ Department of Urology, The Second Affiliated Hospital of Kunming Medical University, Kunming, China

**Keywords:** TP53 mutation, hepatocellular carcinoma, bioinformatics, prognostic model., immune infiltration

## Abstract

**Background:** TP53 mutations are the most frequent mutations in hepatocellular carcinoma (HCC) and affect the occurrence and development of this cancer type. Therefore, it is essential to clarify the function and mechanism of TP53 mutations in HCC.

**Methods:** We performed a sequence of bioinformatic analyses to elucidate the characteristics of TP53 mutations in HCC. We downloaded the data of hepatocellular carcinoma from The Cancer Genome Atlas database and used different R packages for serial analyses, including gene mutation analysis, copy number variation analysis, analysis of the tumor mutational burden and microsatellite instability, differential gene expression analysis, and functional enrichment analysis of TP53 mutations, and performed gene set enrichment analysis. We established a protein-protein interaction network using the STRING online database and used the Cytoscape software for network visualization, and hub gene screening. In addition, we performed anticancer drug sensitivity analysis using data from the Genomics of Drug Sensitivity in Cancer. Immune infiltration and prognosis analyses were also performed.

**Results:** Missense mutations accounted for a great proportion of HCC mutations, the frequency of single nucleotide polymorphisms was high, and C > T was the most common form of single nucleotide variations. TP53 had a mutation rate of 30% and was the most commonly mutated gene in HCC. In the TP53 mutant group, the tumor mutational burden (*p* < 0.001), drug sensitivity (*p* < 0.05), ESTIMATE score (*p* = 0.038), and stromal score (*p* < 0.001) dramatically decreased. The Cytoscape software screened ten hub genes, including CT45A1, XAGE1B, CT55, GAGE2A, PASD1, MAGEA4, CTAG2, MAGEA10, MAGEC1, and SAGE1. The prognostic model showed a poor prognosis in the TP53 mutation group compared with that in the wild-type group (overall survival, *p* = 0.023). Univariate and multivariate cox regression analyses revealed that TP53 mutation was an independent risk factor for the prognosis of HCC patients (*p* <0.05). The constructed prognostic model had a favorable forecast value for the prognosis of HCC patients at 1 and 3 years (1-year AUC = 0.752, 3-years AUC = 0.702).

**Conclusion:** This study further deepened our understanding of TP53-mutated HCC, provided new insights into a precise individualized therapy for HCC, and has particular significance for prognosis prediction.

## Introduction

Hepatocellular carcinoma (HCC) is the second leading cause of cancer-related deaths worldwide (Jiang et al., 2019), as approximately 800,000 HCC-related deaths are expected to occur each year (Rawla et al., 2018). Despite the enormous progress in therapeutic approaches, the HCC recurrence and metastasis rates are still high, leading to an unfavorable prognosis (Li et al., 2018). Statistical analyses (Hou et al., 2019) indicated that the five-year survival rate for HCC is 18%, and only 30–40% of HCC cases are diagnosed at an early stage. In addition, the morbidity and mortality of HCC are still increasing, and HCC is generally considered to be a cancer with a poor prognosis and low survival time. The most important reason is the incomplete understanding of HCC pathogenesis and the lack of clear tumor markers for diagnosis and therapy. Therefore, it is necessary to actively explore and identify valuable HCC diagnostic biomarkers and therapeutic targets that can play an active role in the diagnosis, prevention, and treatment of HCC. This research was designed to explore the pathogenesis of HCC and identify disease-related biomarkers using a series of bioinformatic methods.

The first TP53 mutations were found in colorectal and lung cancer (Baker et al., 1989; Takahashi et al., 1989), and subsequent studies found that TP53 is the most frequently mutated gene in human tumors. A study in which 3281 tumors of 12 cancer types were investigated showed that the frequency of the TP53 mutation was about 40% (Kandoth et al., 2013). Furthermore, mutations of TP53 are the most common type of gene change in HCC, with an average mutation frequency of 30% (Long et al., 2019). In HBV-related HCC patients, this frequency can increase to nearly 60% (Gao et al., 2019; Villanueva, 2019). The wild-type TP53 plays a significant role in cell apoptosis and regulation of the cell cycle after DNA damage (Long et al., 2019). Cells with TP53 protein mutations can escape apoptosis after DNA damage and become cancerous (Wang et al., 2019). This gene maintains the stability of the human genome, and its dysfunction can give rise to centrosome amplification, aneuploid cell proliferation, and chromosomal unsteadiness (Rao et al., 2017). Mutant TP53 protein accumulates in the caryon and is believed to be a highly particular marker of malignancy (Dowell et al., 1994). The mutated TP53 protein not only loses its tumor suppressing function, but also has the ability to promote tumorigenesis (Brosh and Rotter, 2009). The overall survival (OS) and recurrence-free survival (RFS) of HCC patients with TP53 mutations are shorter than those of patients with wild-type TP53 (Liu et al., 2012). The mutation status of TP53 is related to different immune responses (Dong et al., 2017), and studies have proven that the mutation state of TP53 in HCC is connected to the tumor immune microenvironment (Long et al., 2019). The high mutation status of TP53 makes it a potential target for tumor therapy, and anticarcinogens aiming at TP53 mutations are currently in the incipient stages of clinical trials (Cancer Genome Atlas Research Network, 2017). Thus, in-depth elucidation of the impact of TP53 mutations on HCC pathogenesis is essential.

In this study, we performed an extensive analysis of the mutation state and RNA expression of TP53 in HCC to elucidate the biological characteristics of TP53 mutations in HCC. We performed anticancer drug sensitivity analysis, differentially expressed gene analysis, and functional enrichment analysis; constructed a protein-protein interaction (PPI) network; clarified the influence of TP53 mutation on immunological characteristics; and constructed a prognostic model. The results revealed that the immune response of wild-type TP53 was significantly higher than that of mutant TP53. In brief, the mutation status of TP53 and the associated differentially expressed genes can be considered as latent biomarkers and therapeutic targets for HCC.

## Materials and Methods

### RNA-Sequencing Data

We downloaded the “Masked Somatic Mutation” data subjected to VarScan2 analysis from The Cancer Genome Atlas-Liver Hepatocellular Carcinoma (TCGA-LIHC) project on TCGA through the Genomic Data Commons (GDC) data portal (https://portal.gdc.cancer.gov/) as the somatic mutation data of patients with HCC. At the same time, we downloaded patient RNA-seq data (HTSeq-FPKM and counts data) and the corresponding clinical data and converted the level 3 HTSeq-FPKM data into TPM format for subsequent analysis.

### Gene Mutation and Copy Number Variation (CNV)

To analyze the copy number changes in the TP53 gene of TCGA-LIHC patients, we applied the maftools R package (Mayakonda et al., 2018) to analyze and visualize somatic mutations. Meanwhile, GISTIC 2.0 analysis (Reich et al., 2006) was performed on the downloaded CNV fragment using GenePattern5. Finally, the maftools R package was applied to visualize the results.

### Tumor Mutation Burden (TMB) and Microsatellite Instability (MSI)

For each sample, we defined the overall number of somatic mutations detected in the tumor except for silent mutations as the TMB of the sample. MSI refers to the change in microsatellites length in cancer cells because of the insertion or deletion of repeat units relative to normal cells. The TMB and MSI values of each sample were calculated using the maftools and the BiocOncoTK package (Carey et al., 2020).

### Analysis of Anticancer Drug Sensitivity

Genomics of Drug Sensitivity in Cancer (GDSC) is a public database for cancer gene therapy and mutation detection (Vanden Heuvel et al., 2018) (https://www.cancerrxgene.org/). The pRRophetic package (Geeleher et al., 2014) was used to download cell line gene mutation data and the IC50 values of different anticancer drugs, and to study the correlation between TP53 mutation and anticancer drug sensitivity.

### Differential Gene Expression and Functional Enrichment

According to the mutations of TP53 in TCGA-LIHC, data were split into mutant and wild-type groups, and the DESeq2 package (Love et al., 2014) was used to execute differential gene expression analysis. Log FC >1.5 and an adjusted *p*-value <0.05 were set as the threshold values for recognizing differentially expressed genes. The clusterProfiler R (Subramanian et al., 2005) package was used for Gene Ontology (GO) annotation and Kyoto Encyclopedia of Genes and Genomes (KEGG) pathway analysis of the differentially expressed genes.

### Gene Set Enrichment Analysis (GSEA)

GSEA starts with the expression matrix of genes, divides them into two groups on the grounds of gene expression or mutation, and predicts the signal pathways that may be associated with the gene set by analyzing the differences in signal pathway enrichment between the two groups (Subramanian et al., 2005). To investigate the differences in biological characteristics between different groups, the “hall.v7.2.symbols.gmt (Hallmarks)” gene set was downloaded from the MSigDB database for GSEA analysis, and *p* <0.05 was considered statistically significant.

### Construction of PPI Network

The STRING database (Szklarczyk et al., 2019) is an online tool for studying the interaction network between proteins (https://string-db.org/). Genes with a score >0.4 were screened based upon the interaction of the STRING database. The interaction network was constructed and visualized utilizing the Cytoscape software (Shannon et al., 2003); the top ten genes were obtained by each of the 12 algorithms (Betweenness, BottleNeck, Closeness, ClusteringCoefficient, Degree, DMNC, EcCentricity, EPC, Maximal Clique Centrality (MCC), MNC, Radiality, Stress) in the CytoHubba plugin (Chin et al., 2014), and then intersected and visualized by applying UpsetR.

### Immune Infiltration Analysis of TP53 Mutations

ESTIMATE analysis quantifies the immune infiltration level in a tumor sample based on the gene expression profile. We evaluated the immune activity of each tumor sample and its matrix score using the ESTIMATE package (Yoshihara et al., 2013). The Euclidean cluster analysis approach was applied to analyze the classification of TP53 mutations, immune genes, and Human Leukocyte Antigen (HLA) gene families. Furthermore, we used CIBERSORT (Newman et al., 2015) to appraise the abundance of immune cells in TCGA-LIHC and used the “Cibersort R” package to confirm the ratio of 22 tumor-infiltrating immune cells (TIICs) in each sample. TIICs included naive B cells (Bn), B cell memory (Bm), plasma cells, CD8^+^ T cells, naïve CD4^+^ T cells (CD4^+^ Tn), resting memory CD4^+^ T cells (CD4^+^ Tmr), activated memory CD4^+^ T cells (CD4^+^ Tma), follicular helper T cells (Tfh), regulatory T cells (Tregs), T cells gamma delta (γδT), natural killer cells resting (NKr), natural killer cells activated (NKa), monocytes, M0 macrophages (M0), M1 macrophages (M1), M2 macrophages (M2), resting dendritic cells (DCr), activated dendritic cells (DCa), resting mast cells (Mr), activated mast cells (Ma), eosinophils, and neutrophils.

### Construction of Prognostic Model

The prognostic factors of TCGA-LIHC were evaluated utilizing Cox regression analysis and the Kaplan-Meier approach. We used the rms package (Eng et al., 2015) to draw the nomogram and calibration curve. The nomogram shows a prognostic prediction model and provides a scoring tool to evaluate risk probability. The calibration curve shows the consistency between the risk possibility forecasted by the nomogram and the actual possibility and evaluates the effectiveness of the prognostic model. Moreover, the time-dependent receiver operating characteristic (ROC) curve was established utilizing the time-ROC package (Blanche et al., 2013), the area under the curve (AUC) was computed, and the exactitude of the risk score was assessed to estimate the prognosis.

### Statistical Analysis

For the statistical analysis of two groups of normally distributed continuous variables, the t-test was used. The Wilcoxon rank sum test was used for data with a skewed distribution. Categorical variables were disposed utilizing the chi-square test or Fisher’s exact test. All *p*-values were two-sided, and statistical significance was set at *p* < 0.05.

## Results

### TP53 Mutation and CNV in TCGA-LIHC

**FIGURE 1:**
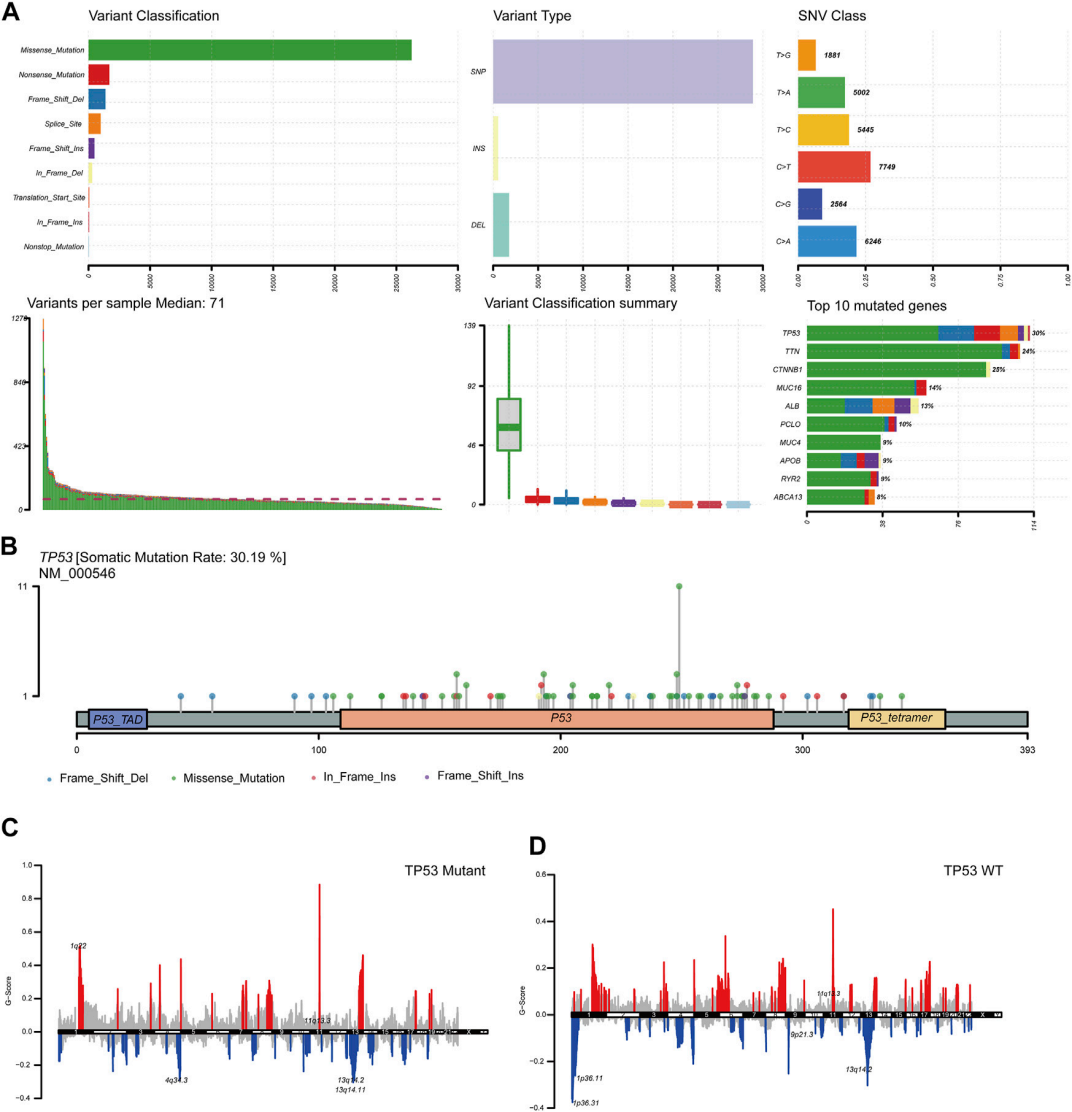
TP53 gene mutation and copy number variation in LIHC patients. **(A)**: Summary of mutation information in LIHC patients. Missense mutations account for the majority of different mutation types; SNP frequency is higher than insertion or deletion, and C > T is the most common mutation of SNV; tumor mutation burden in specific samples and the top 10 mutated genes. **(B)**: Distribution map of amino acid variation of TP53 protein in TCGA-LIHC. **(C,D)**: GISTIC2.0 copy number variation analysis results, red represents copy number amplification, blue represents copy number deletion.

To analyze the impact of TP53 mutations on LIHC patients, we downloaded patient mutation data from the TCGA database. First, we analyzed the overall mutation status of TCGA-LIHC, and the results showed that missense mutations accounted for the main part, single nucleotide polymorphisms (SNP) occurred more frequently than insertions or deletions, and C > T was the most common type of single nucleotide variants (SNVs) in LIHC patients; the TMB in a specific sample and the supreme ten mutated genes were also determined (Figure 1A). Analysis of the amino acid changes in TP53 showed that missense mutations were the main mutation type (Figure 1B). Simultaneously, based on the mutation status of TP53, we divided the CNV data of TCGA-LIHC into TP53 mutation and wild-type groups, and found that both TP53-MT and TP53-WT groups had a large CNV regardless of the states of TP53. The significantly amplified regions in TP53-MT group included 1q22 and 11q13.3, and significant deletions in the TP53-MT group included 4q34.3, 13q14.2, and 13q14.11 (Figures 1C,D).

### Biological Characteristics of TP53 Mutations

**FIGURE 2:**
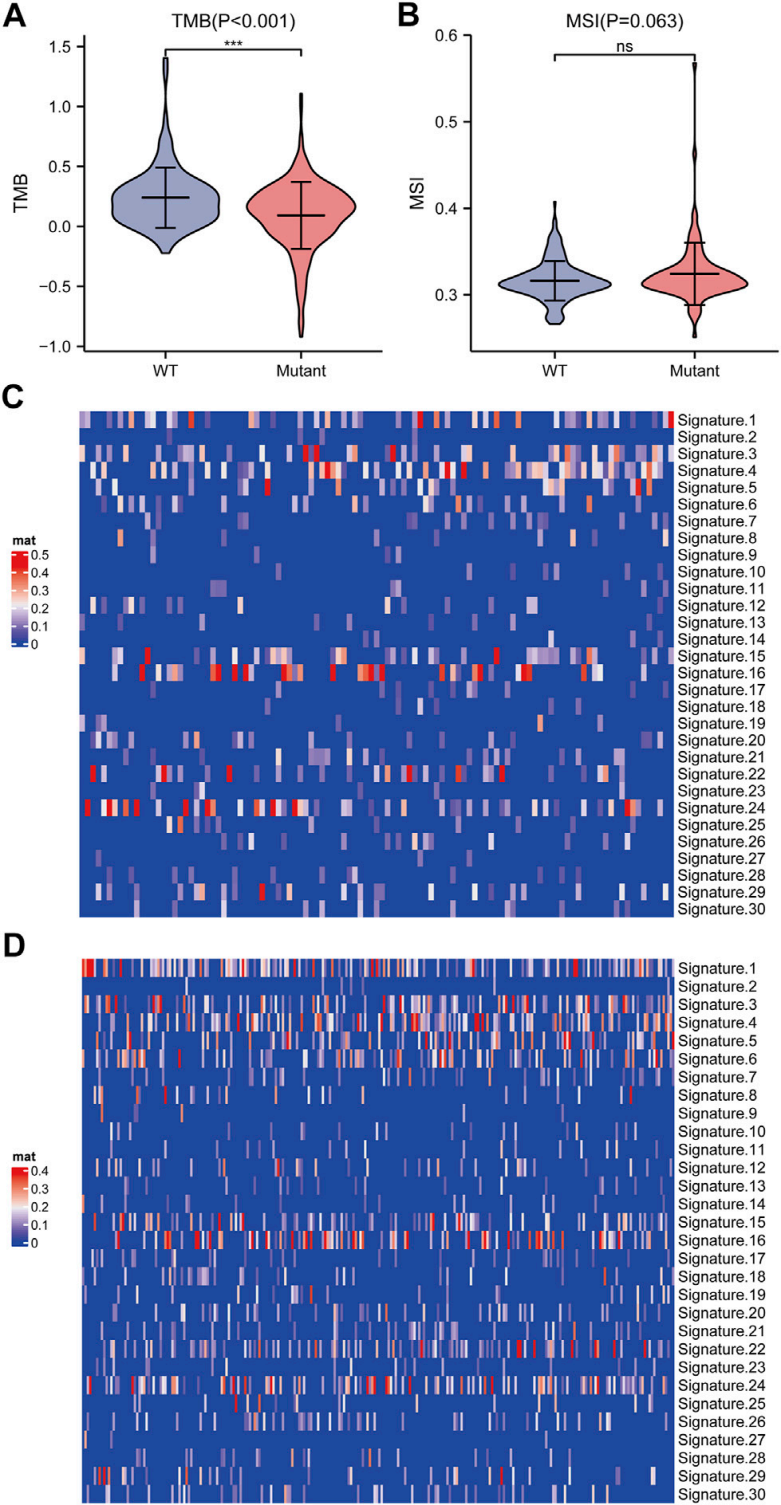
Analysis of biological characteristics of TP53 gene mutation level. **(A)**: TMB in patients with TP53 mutations was significantly reduced (*p* <0.001). **(B)**: There is no significant difference in MSI (*p* = 0.063). **(C)**: Cosmic Signature heat map of TP53 mutation patients. **(D)**: Cosmic Signature heat map of TP53 non-mutant patients.

**FIGURE 3:**
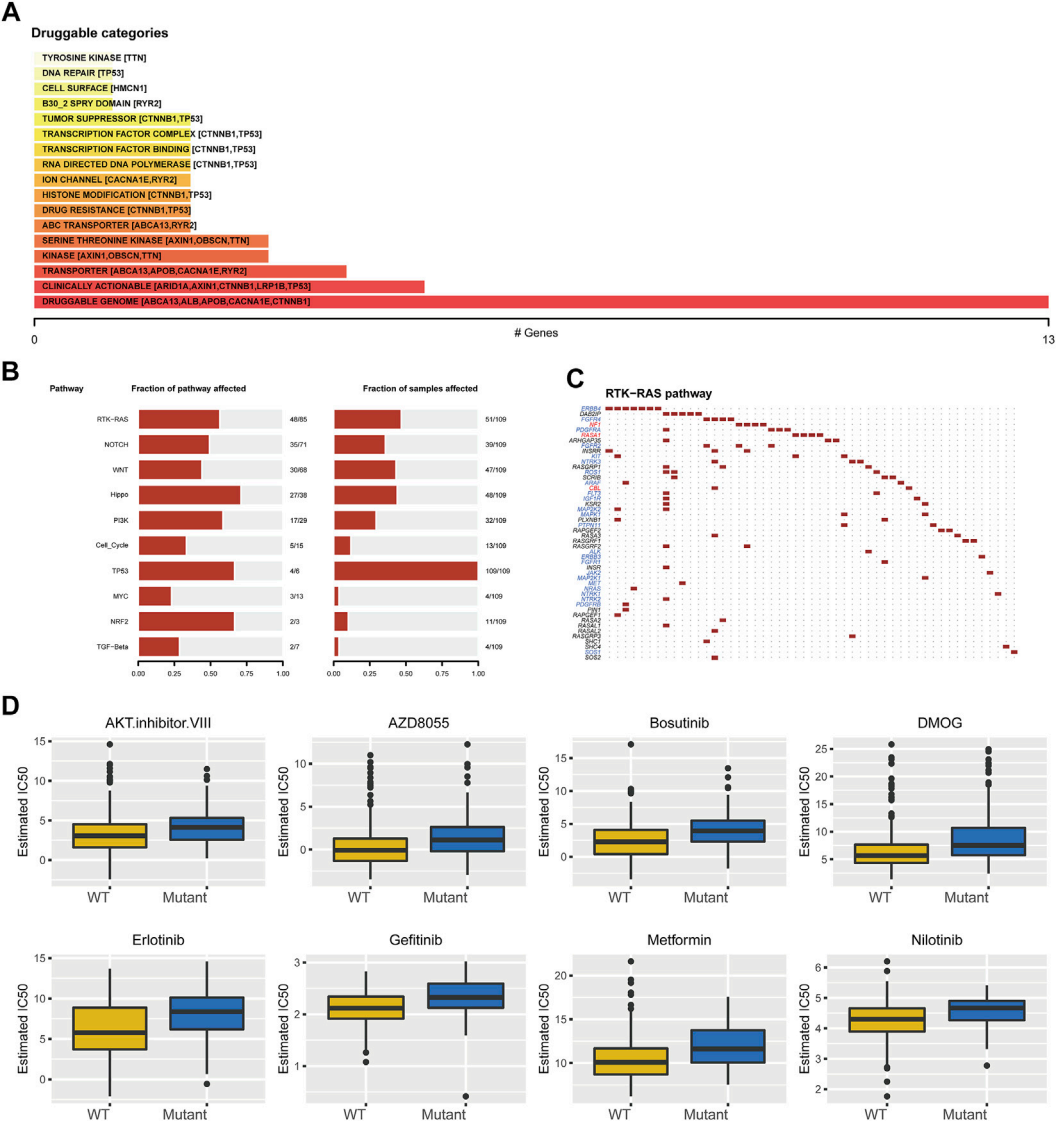
Drug sensitivity analysis of TP53 gene mutation. **(A)**: The relationship between gene mutation level in TCGA-LIHC and different kinds of drugs. **(B)**: Changes of gene mutation levels in different carcinogenic signal pathways. **(C)**: Mutation distribution of main genes in RTK-Ras signaling pathway. **(D)**: Sensitivity of TP53 mutation to different chemotherapeutics and small molecule anticancer drugs.

We further elucidated the influences of TP53 mutations on different biological characteristics. The TMB of the TP53 mutation group was prominently inferior to that of the wild-type group (*p* <0.001; Figure 2A). MSI was not notably different between the TP53 mutation and wild-type groups (*p* = 0.063; Figure 2B). Furthermore, Sanger decomposed 96 mutation spectra into 30 different signatures based on somatic mutations combined with biological characteristics (Bertucci et al., 2019). The results indicated that the signature distributions of the two groups were not significantly different (Figures 2C,D). Furthermore, we discovered a correlation between multiple drugs and the gene mutation levels in TCGA-LIHC, especially the druggable genome (Figure 3A). Pathway analysis indicated that the RTK-Ras pathway was enriched in the TP53 mutation group (Figure 3B), and related genes in the RTK-Ras pathway had a high mutation rate in LIHC patients (Figure 3C). To analyze the influence of gene mutations on drug sensitivity, we downloaded cell line gene mutation data and the IC50 values of different anticancer drugs from the GDSC database. According to the cell line responsiveness to 138 different chemotherapeutic drugs and small molecule anticancer drugs, the IC50 values of anticancer drugs in TCGA-LIHC patients were predicted. The results revealed that the IC50 values of 64 chemotherapeutics and small molecule anticancer drugs were significantly different between patients with TP53 mutation and those with wild-type TP53 (*p* <0.05; Figure 3D), including the AKT inhibitor VIII, AZD8055, bosutinib, DMOG, erlotinib, gefitinib, metformin, nilotinib, and other drugs.

### Differential Gene Expression and Functional Enrichment Analysis

**FIGURE 4:**
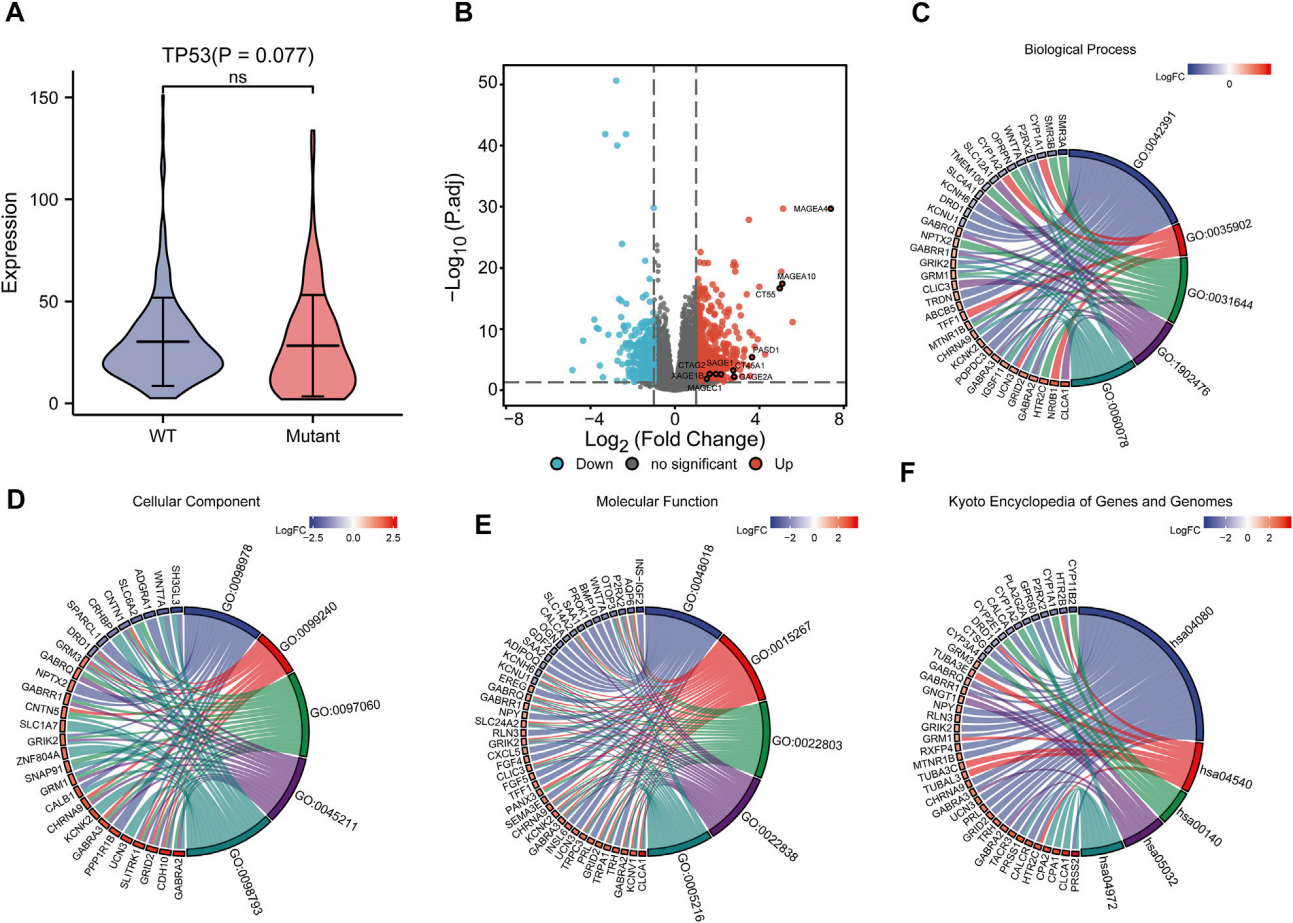
Differential expression and functional enrichment analysis based on TP53 gene mutation. **(A)**: There was no significant difference in the expression level between TP53 mutation and non-mutation (*p* = 0.077). **(B)**: Volcano map of differentially expressed genes between TP53 mutant and non-mutant groups. GO analysis of differentially expressed genes, **(C)**: Biological processes, **(D)**: cellular component, **(E)**: molecular function. **(F)**: KEGG pathway enrichment analysis.

**FIGURE 5:**
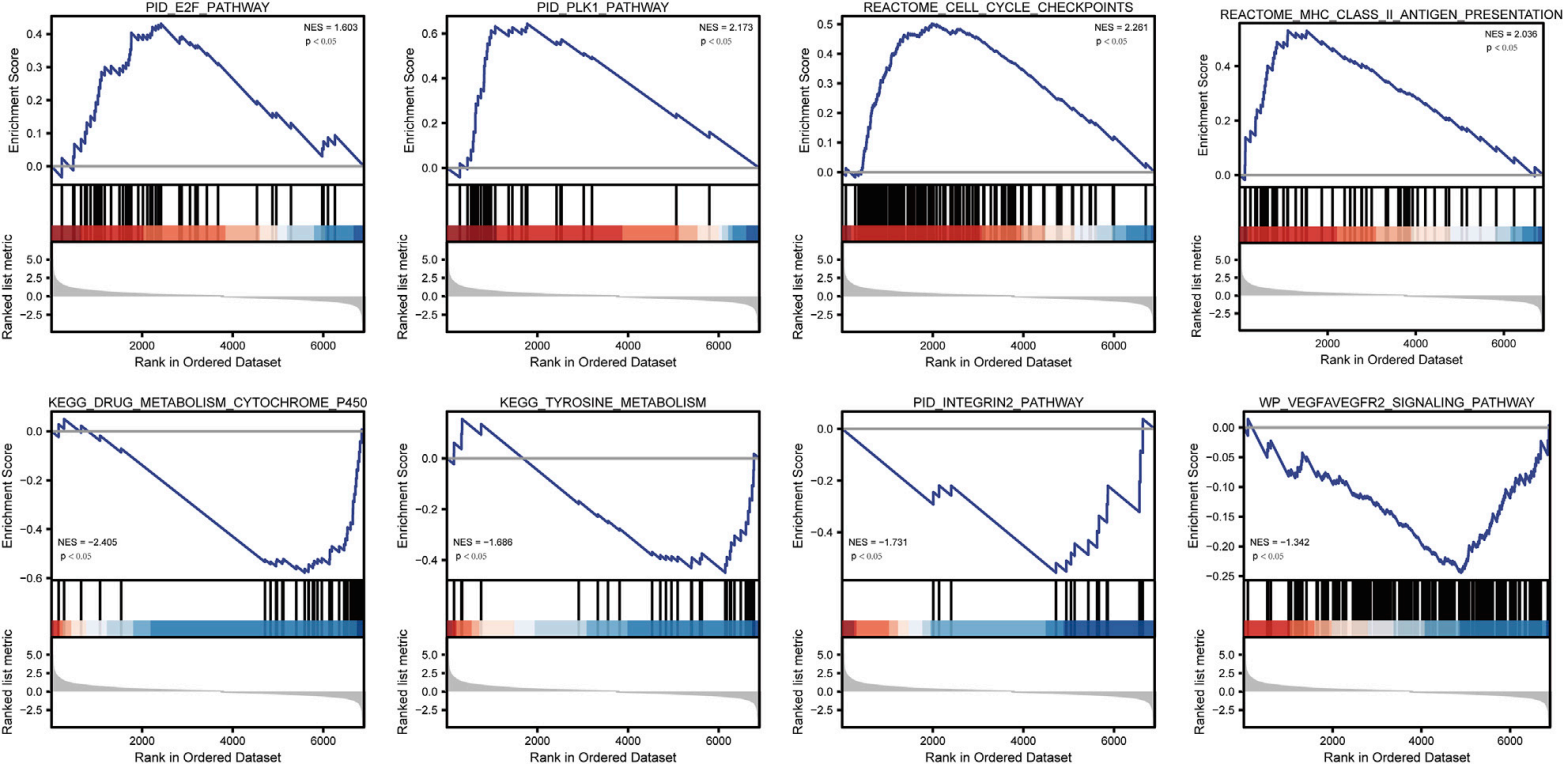
GSEA analysis.

To analyze the effect of TP53 mutation on HCC tumorigenesis, based on the mutation of TP53, we distributed the patients into the P53 mutation and wild-type groups. The results showed that the overall gene expression level was not remarkably different between the TP53 mutation and wild-type groups (Figure 4A). Subsequently, we conducted differential gene expression analysis of the two groups and found that the expression of 256 genes was significantly up-regulated, and that of 111 genes was significantly down-regulated in the TP53 mutation group (Figure 4B). GO analysis results (Table 1) revealed that the differentially expressed genes were closely related to biological processes, such as regulation of membrane potential, response to immobilization stress, administration of neurological system process, chloride transmembrane transport, regulation of postsynaptic membrane potential, and the chemokine-mediated signaling pathway (Figure 4C). Cell components were mainly enriched in glutamatergic synapses, intrinsic components of synaptic membranes, synaptic membranes, postsynaptic membranes, and presynapses (Figure 4D). Molecular functions were chiefly enriched in receptor ligand activity, channel activity, passive transmembrane transporter activity, substrate-specific channel activity, and ion channel activity (Figure 4E). KEGG analysis showed that it mainly affected neuroactive ligand-receptor interaction, gap junction, steroid hormone biosynthesis, morphine addiction, and the pancreatic secretion signaling pathways (Figure 4F; Table 2). The GSEA results showed (Figure 5; Table 3) that the PID-E2F-pathway, PID-PLK1-pathway, reactome-cell-cycle-checkpoints, and reactome-MHC-CLASS-II-antigen-presentation were significantly enriched in the TP53 mutation group. The TP53 wild-type group was closely related to KEGG-drug-metabolism-cytochrome-P450, KEGG-tyrosine-metabolism, the PID-integrin2-pathway, and the WP-VEGFAVEGFR2-signaling-pathway.

**TABLE 1:** GO analysis.

Ontology	ID	Description	Generatio	*P* value
BP	GO:0042391	Regulation of membrane potential	20/297	2.27e-05
BP	GO:0035902	Response to immobilization stress	5/297	5.97e-05
BP	GO:0031644	Regulation of neurological system process	10/297	6.91e-05
BP	GO:1902476	Chloride transmembrane transport	8/297	7.97e-05
BP	GO:0060078	Regulation of postsynaptic membrane potential	10/297	8.29e-05
CC	GO:0098978	Glutamatergic synapse	15/327	7.75e-04
CC	GO:0099240	Intrinsic component of synaptic membrane	9/327	0.002
CC	GO:0097060	Synaptic membrane	16/327	0.002
CC	GO:0045211	Postsynaptic membrane	13/327	0.003
CC	GO:0098793	Presynapse	17/327	0.004
MF	GO:0048018	Receptor ligand activity	22/289	1.51e-05
MF	GO:0015267	Channel activity	21/289	2.08e-05
MF	GO:0022803	Passive transmembrane transporter activity	21/289	2.15e-05
MF	GO:0022838	Substrate-specific channel activity	20/289	2.67e-05
MF	GO:0005216	Ion channel activity	18/289	1.77e-04

**TABLE 2:** KEGG analysis.

Ontology	ID	Description	GeneRatio	BgRatio	*P* value	*P* adjust	Q value
KEGG	hsa04080	Neuroactive ligand-receptor interaction	27/121	341/8076	5.18e-13	8.65e-11	7.96e-11
KEGG	hsa04540	Gap junction	7/121	88/8076	3.31e-04	0.028	0.025
KEGG	hsa00140	Steroid hormone biosynthesis	5/121	61/8076	0.002	0.066	0.060
KEGG	hsa05032	Morphine addiction	6/121	91/8076	0.002	0.066	0.060
KEGG	hsa04972	Pancreatic secretion	6/121	102/8076	0.004	0.087	0.080

**TABLE 3:** GSEA analysis.

Description	Enrichmentscore	NES	*P* value
REACTOME_CELL_CYCLE_CHECKPOINTS	0.501341175	2.261135055	0.001222494
PID_PLK1_PATHWAY	0.643570445	2.172850425	0.001501502
REACTOME_MHC_CLASS_II_ANTIGEN_PRESENTATION	0.530972657	2.036020176	0.001420455
PID_E2F_PATHWAY	0.430807754	1.603065605	0.014492754
WP_VEGFAVEGFR2_SIGNALING_PATHWAY	−0.24403672	−1.341828375	0.029761905
KEGG_TYROSINE_METABOLISM	−0.449066106	−1.685998834	0.014492754
PID_INTEGRIN2_PATHWAY	−0.556102027	−1.731321158	0.024449878
KEGG_DRUG_METABOLISM_CYTOCHROME_P450	−0.577290478	−2.405141843	0.003067485

### Construction of PPI Network and Hub Gene Recognition

**FIGURE 6:**
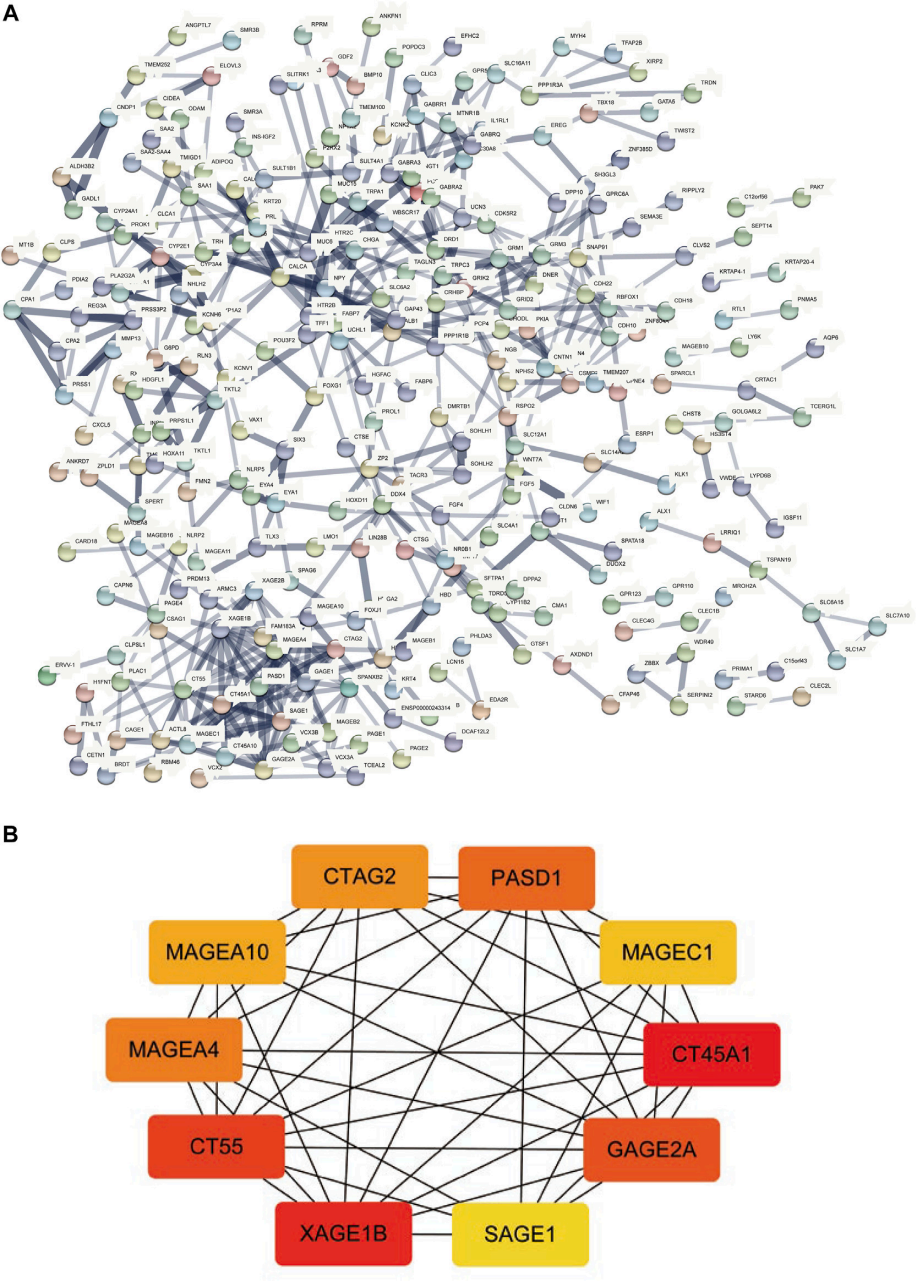
PPI network construction and hub-gene screening. **(A)**: PPI network of differentially expressed genes. **(B)**: Get the top 10 genes according to the MCC algorithm, the redder the color, the more important.

The STRING database was utilized for the PPI network of differentially expressed genes (Figure 6A), and the interaction relationship was imported into the Cytoscape software. The top ten genes were obtained by each of the 12 algorithms in the CytoHubba plugin and then intersected and visualized by applying UpsetR (Supplementary Figure S1). However, no genes were detected after taking the intersection. We used the MCC algorithm to select the ten genes with the highest expression as hub genes, which included CT45A1, XAGE1B, CT55, GAGE2A, PASD1, MAGEA4, CTAG2, MAGEA10, MAGEC1, and SAGE1 (Figure 6B).

### Immunological Characteristics of TP53 Mutations

**FIGURE 7:**
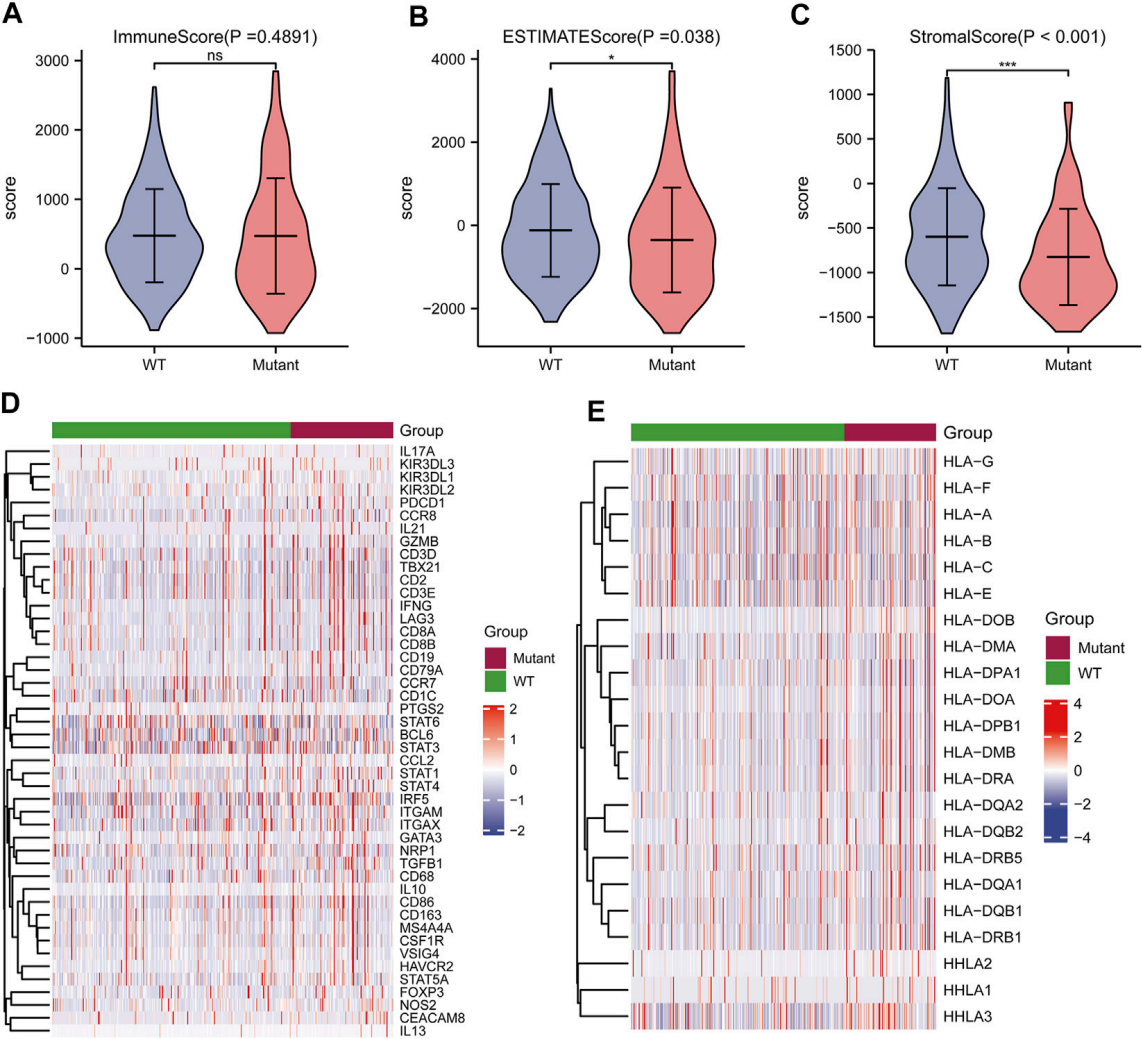
Effect of TP53 mutation on the immunological characteristics of TCGA-LIHC patients. **(A)**: ImmuneScore has no significant difference (*p* = 0.4891). **(B)**: ESTIMATE Score decreased significantly (*p* = 0.038). **(C)**: StromalScore was significantly reduced (*p* <0.001). **(D)**: Cluster heat map of TP53 mutation and immune gene. **(E)**: Cluster heat map of TP53 mutation and HLA gene family.

**FIGURE 8:**
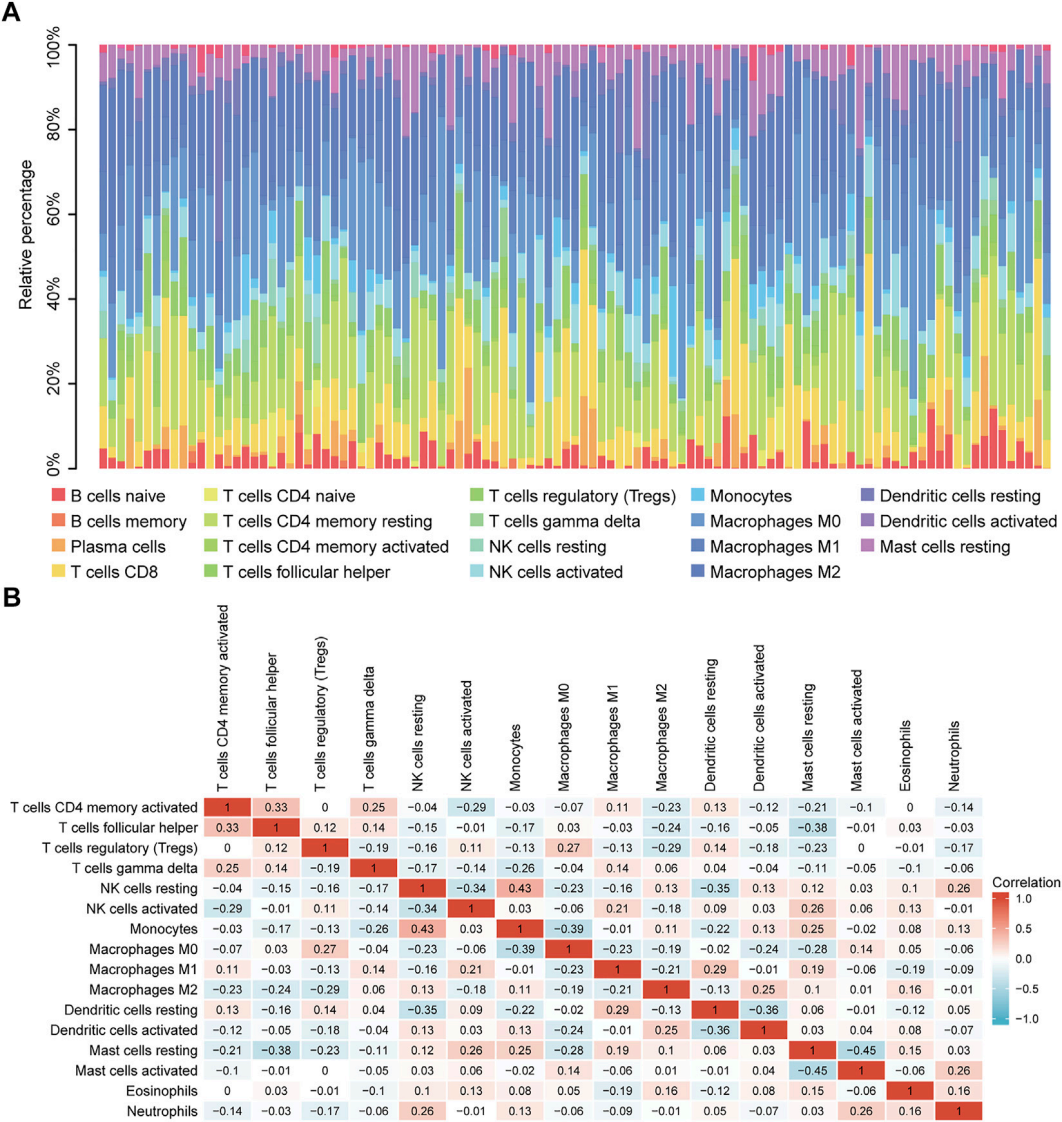
CIBERSORT analysis. **(A)**: Abundance of immune cells in TP53 mutant TCGA-LIHC samples. **(B)**: Correlation heat map between different immune cell infiltration.

Correlation analysis between TP53 mutations and TCGA-LIHC immunological characteristics revealed that the immune score of the wild-type group was not significantly different from that of the TP53 mutation group (*p* = 0.4891, Figure 7A), while the ESTIMATE score (*p* = 0.038, Figure 7B), and the stromal score were significantly reduced (*p* <0.001, Figure 7C). The cluster heat maps of TP53 with immune genes and HLA family genes are shown in Figures 7D,E. In addition, we applied CIBERSORT to evaluate the abundance map of immune cells in TP53 mutant TCGA-LIHC samples (Figure 8A) and the correlation between different immune cell infiltrations (Figure 8B).

### Construction of Prognostic Model

**FIGURE 9:**
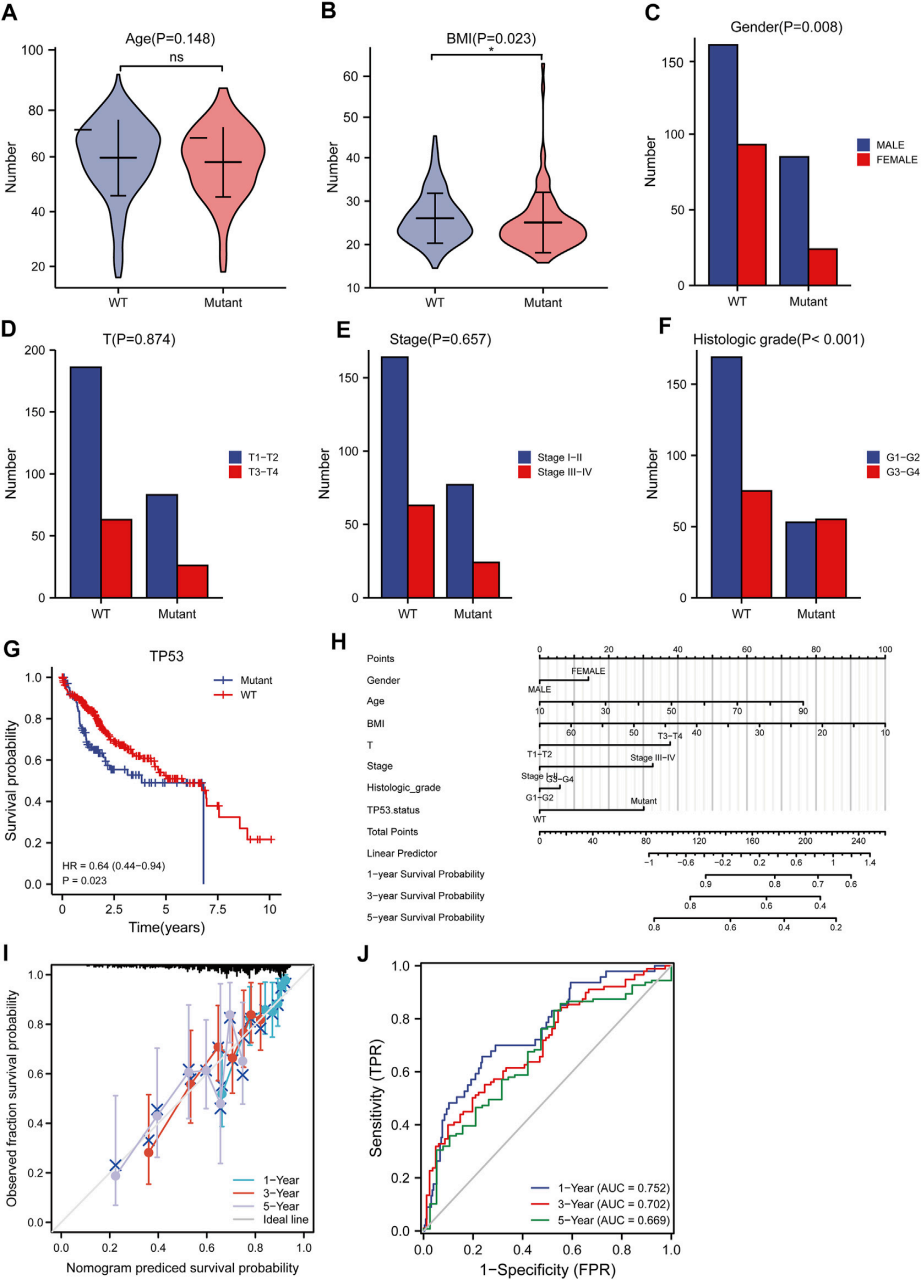
Analysis of clinical correlation of TP53 mutation and construction of prognostic model. **(A)**: There was no significant difference in age between the TP53 mutation group and the non-mutation group (*p* = 0.148). **(B)**: The BMI of the TP53 mutation group was significantly low (*p* = 0.023). **(C)**: There is a significant difference in gender (*p* = 0.008). **(D,E)**: No significant difference in clinical T staging (*p* >0.05). **(F)**: There are significant differences in pathological grading (*p* <0.001). **(G)**: Survival analysis showed that OS with TP53 mutation was significantly shortened (*p* = 0.023). **(H)**: nomogram. **(I)**: Calibration curve. **(J)**: Time-dependent ROC curve.

The baseline data of patients with TP53 mutation and those with wild-type TP53 are shown in Table 4. Relative to those in the wild-type group, the age and stage of the patients in the TP53 mutation group were not statistically significant (*p* > 0.05), but there were remarkable differences in the body mass index (BMI), sex, and pathological grade (*p* < 0.05, Figures 9A–F). Survival analysis indicated that the TP53 mutation group had a poor prognosis in LIHC (overall survival, *p* = 0.023, Figure 9G). Univariate and multivariate Cox regression analysis results showed that TP53 was an independent prognostic risk factor for LIHC patients (*p* < 0.05, Table 5). We then constructed a prognostic model based on the above clinical features and drew a nomogram to evaluate the risk probability (Figure 9H). In addition, the calibration curve (Figure 9I) and time-dependent ROC (Figure 9J) indicated that the model had a relatively favorable predictive value for the prognosis of patients at 1 and 3 years (1-year AUC = 0.752, 3-years AUC = 0.702).

**TABLE 4:** Baseline data.

Characteristic	TP53-WT	TP53-mutant	*p*
N	252	109	—
Gender, n (%)	—	—	0.008
Female	93 (25.8%)	24 (6.6%)	—
Male	159 (44%)	85 (23.5%)	—
T, n (%)	—	—	0.874
T1–T2	186 (52%)	83 (23.2%)	—
T3–T4	63 (17.6%)	26 (7.3%)	—
Stage, n (%)	—	—	0.657
Stage I–II	175 (51.5%)	77 (22.6%)	
Stage III–IV	64 (18.8%)	24 (7.1%)	
Histologic_grade, n (%)	—	—	<0.001
G1–G2	171 (48%)	53 (14.9%)	—
G3–G4	77 (21.6%)	55 (15.4%)	—
Age, median (IQR)	61 (52, 69)	59 (50, 67)	0.148
BMI, median (IQR)	25.18 (21.94, 29.19)	23.52 (20.97, 27)	0.023

**TABLE 5:** Univariate and multivariate Cox analysis

Characteristics	Total (*n*)	Univariate analysis		Multivariate analysis	
		Hazard ratio (95% CI)	*p* value	Hazard ratio (95% CI)	*p* value
Gender	376	—	—	—	—
Male	254	References	—	—	—
Female	122	1.218 (0.856–1.733)	0.272	—	—
Age	376	1.013 (0.999–1.027)	0.066	1.014 (0.999–1.029)	0.067
BMI	340	0.974 (0.943–1.007)	0.121	—	—
T	373	—	—	—	—
T1–T2	279	References	—	—	—
T3–T4	94	2.502 (1.762–3.553)	<0.001	1.604 (0.218–11.785)	0.642
Stage	352	—	—	—	
Stage I–II	261	References	—	—	
Stage III–IV	91	2.409 (1.666–3.484)	<0.001	1.587 (0.218–11.576)	0.649
histologic_grade	371	—	—	—	—
G2	180	References	—	—	—
G1	55	0.851 (0.503–1.439)	0.547	—	—
G3	123	1.021 (0.692–1.508)	0.915	—	—
G4	13	1.265 (0.507–3.159)	0.615	—	—
TP53.status	360	—	—	—	—
WT	251	References	—	—	—
Mutant	109	1.558 (1.063–2.285)	0.023	1.601 (1.066–2.404)	0.023

## Discussion

HCC is the most widespread hypotype of liver cancer and is one of the principal causes of cancer-related mortality worldwide (Torre et al., 2015). Because of the dormant symptoms and early metastasis, the majority of HCC patients are diagnosed at the terminal stage, leading to inferior efficacy or even invalidity of therapeutic methods (Villanueva et al., 2010). Although diagnostic and treatment approaches have significantly improved, the prognosis of HCC patients remains poor. Hence, it is essential to identify the underlying mechanisms and diagnostic biomarkers for HCC. Furthermore, the identification of promising therapeutic targets is crucial.

Carcinogenesis is a sophisticated multi-step, multi-stage process involving both genetic and epigenetic variations (Villanueva et al., 2010). In the last ten years, there has been an explicit characterization of the landscape of genetic changes in HCC (Zucman-Rossi et al., 2015), and TP53 is one of the most commonly mutated genes in HCC. Studies have confirmed that TP53 can be used as a biomarker of some molecular characteristics and is a poor prognostic factor in HCC (Wen et al., 2016; Long et al., 2019). Nevertheless, the mechanism by which TP53 mutations affect the occurrence and development of HCC, immune phenotypic regulation, and poor prognosis is currently unclear.

This study provides a new understanding of the pathogenesis of HCC by presenting a comprehensive analysis of TP53-mutated HCC. We analyzed somatic mutations in HCC and found that missense mutations accounted for the main part of HCC mutations, and the frequency of SNPs was higher than that of insertions or deletions. Furthermore, the top ten mutant genes were identified, including TP53, TTN, CTNNB1, MUC16, ALB, PCLO, MUC4, APOB, RYR2, and ABCA13. The CNV levels of multiple genes in the TP53 mutant group were prominently different from those of the wild-type group. The TMB, anticancer drug sensitivity, immune score, and immune cell abundance of the TP53 mutation group decreased compared with those of the wild-type group. We divided patients into mutated and wild-type groups according to the TP53 mutation status, analyzed differentially expressed genes, and performed GO and KEGG analyses. A PPI network of differentially expressed genes was constructed, and hub genes, including CT45A1, XAGE1B, CT55, GAGE2A, PASD1, MAGEA4, CTAG2, MAGEA10, MAGEC1, and SAGE1 were identified.

CT45A1 is a cancer/testis antigen family 45 member A1, which has the ability to promote tumorigenesis and metastasis in osteosarcoma and breast cancer (Shang et al., 2014; Wen et al., 2021). It has been reported that CT45A1 plays a cancer-promoting role by triggering epithelial-mesenchymal transition (EMT) and cancer stem cell generation (Yang et al., 2015). Silencing of CT45A1 in lung cancer cells inhibited tumor cell proliferation, invasion and metastasis (Tang et al., 2017). XAGE1B is a X antigen family member 1B, and also belongs to the cancer-testis antigen family. It is highly expressed in HCC and related to the invasive biological behavior of HCC cells, but the specific mechanism is not fully understood (Pan et al., 2014). CT55 is also a member of the cancer testicular antigen family, and studies have found that CT55 deficiency inhibits the NF-KB signaling pathway, thereby attenuating colitis-associated cancers (Zhao et al., 2019). Studies have also shown that CT55 regulates mitochondrial activity and thus maintains the proliferation of cancer cells (Aurrière et al., 2022). The screened hub gene plays an active role in a variety of tumors, but the relationship with TP53 in HCC has not been reported, which provides a new direction for future studies.

Survival analysis revealed that patients with TP53 mutations had a bad prognosis. Cox regression analysis showed that TP53 was an independent prognostic risk factor for HCC. The prognostic model constructed according to the clinical data of patients had a relatively favorable predictive value for 1-year and 3-years prognoses. Moreover, the results of this research may indicate a viable therapeutic strategy targeting the immune microenvironment to improve the prognosis of HCC.

Cancers have a variety of immunosuppressive mechanisms according to the tumor immunoediting hypothesis, which enable tumor cells to evade the anti-tumor immune response during cancer development, and an immunologically active host selects cancer cells with lower immunogenicity (immune selection) and establishes an immunosuppressive network (immune evasion) (Dunn et al., 2002; Schreiber et al., 2011). Cancers have the ability to increase the abundance of immunosuppressive cells (Treg cells and tumor-associated macrophages) and the expression of immunosuppressive molecules (cytotoxic T lymphocyte-associated antigen 4, CTLA-4), and reduce the expression of tumor antigens, resulting in CD8^+^ T cells being unable to identify cancer cells (Zou and chen, 2008; Pardoll 2012). Our study indicates that the TMB, immune score, and immune cell abundance in TP53 mutant HCC are reduced, and that TP53 mutations may also cause tumorigenesis and a poor prognosis through tumor immunosuppressive mechanisms. The total number of somatic gene coding errors, base substitutions, gene insertions, or deletions detected per mega base is referred to as the TMB, and tumors with high TMB have more neoantigens, which can help the immune system recognize the tumor and stimulate T cell proliferation and anti-tumor responses. The decrease in the TMB and immune score in the TP53 mutant group may be the reason for the decreased drug sensitivity relative to that of the TP53 wild-type group, which ultimately leads to a poorer prognosis for the TP53 mutant group. Thus, blocking the immunosuppressive mechanism enables to restore the potential antitumor immune response and achieve the goal of tumor treatment. However, in breast cancer, the TP53 mutant group exhibited higher TMB values (*p* < 0.001) and a significantly higher stromal score (*p* = 0.003); by contrast, KEGG and GSEA enrichment analyses of TP53 mutant breast cancer yielded different results (Zhang et al., 2021). This shows that TP53 mutations exhibit different characteristics in different tumors. In addition, univariate and multivariate Cox analyses suggested that TP53 mutation is an independent risk factor for the prognosis of HCC, which may provide a novel biomarker for the prediction of disease status and prognosis of HCC patients, which is extremely vital for appropriate monitoring and selection of treatment methods. Although the wild-type TP53 protein is promptly degraded in an MDM-dependent mode and can thus not be detected, the mutated TP53 protein can evade degradation and accumulates to detectable levels in the nucleus (Midgley and Lane, 1997). In addition, the mutated TP53 protein can be released from HCC cells into the blood, thereby inducing the production of detectable anti-TP53 antibodies (Volkmann et al., 1993). The level of serum anti-TP53 antibodies can theoretically reflect the cumulation of mutant TP53 protein in cancer cells, which has a certain role in diagnosis and prognosis, although further study is required to verify this. Our research provides novel directions for the diagnosis and treatment of HCC. TP53 mutations may potential pathogenic factors and therapeutic targets in HCC.

Nevertheless, this research had some limitations. First, the sample size of the analysis was relatively small, and the statistical power may be insufficient. Therefore, further studies with a greater sample size and a prospective design are required to improve the statistical efficiency and obtain more meaningful results. Second, the results of this research are based upon bioinformatic analysis and have certain limitations, which require further experimental and clinical verification. Finally, the average frequency of TP53 mutations in HCC is approximately 30%, and most HCC patients were not within the scope of this research. Further studies need to be conducted to represent more HCC patients.

In summary, this study analyzed gene mutations in HCC, conducted a series of analyses on HCC with TP53 mutations, and constructed a prognostic model. Overall, we provide new insights into the individualized and precise prediction and treatment of HCC.

## Data Availability

The datasets for this study can be found in the TCGA database (https://portal.gdc.cancer.gov/) and GDSC database (https://www.cancerrxgene.org/).

## Abbreviations

AUC, area under curve; BMI, body mass index; CNV, copy number variation; GDSC, genomics of drug sensitivity in cancer; GO, gene ontology; GSEA, gene set enrichment analysis; HCC, hepatocellular carcinoma; KEGG, kyoto encyclopedia of genes and genomes; LIHC, liver hepatocellular carcinoma; MCC, maximal clique centrality; MSI, microsatellite instability; MSigDB, molecular signatures database; OS, overall survival; PPI, protein-protein interaction; RFS, recurrence-free survival; ROC, receiver operating characteristic; SNP, single nucleotide polymorphisms; SNV, single nucleotide variant; TCGA, the cancer genome atlas; TMB, tumor mutational burden.
