# Supplementary figures and images for: Identification of Novel Characteristics in TP53-Mutant Hepatocellular Carcinoma Using Bioinformatics
Source: Front Genet. 2022 May 16;13:874805. doi: 10.3389/fgene.2022.874805 (PMC9149291; doi:10.3389/fgene.2022.874805)

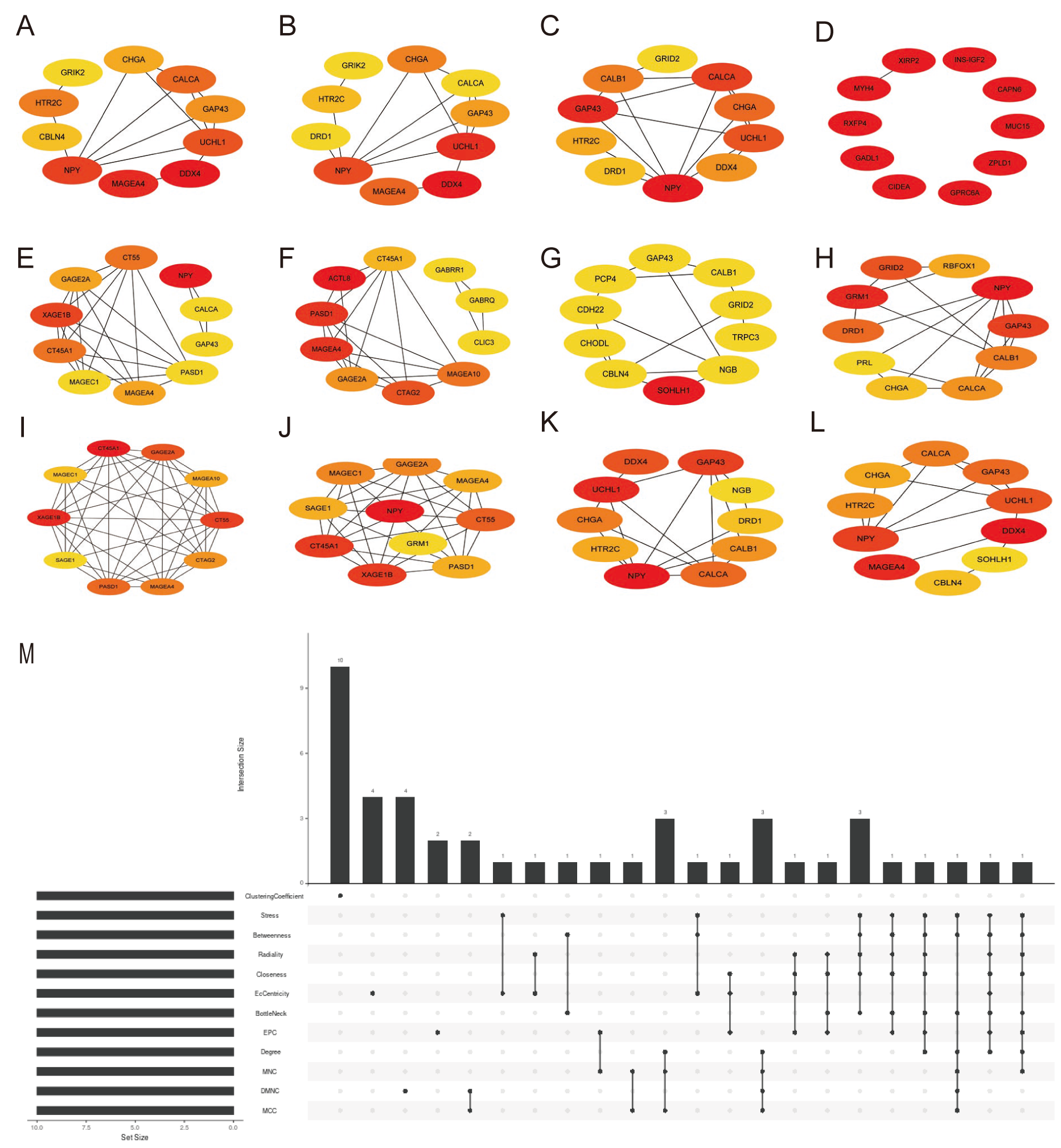

Supplement: Supplementary file 1 [file Image1.TIF]
